# Experiments to Determine the Antiseptic Value of Boric Acid

**Published:** 1889-03

**Authors:** Edmund Andrews

**Affiliations:** Professor of Clinical Surgery in the Chicago Medical College; 6 Sixteenth Street


### CHICAGO MEDICAL JOURNAL AND EXAMINER.

VOLUME LVIII. MARCH, 1889. NUMBER 3.

### *EXPERIMENTS TO DETERMINE THE ANTISEPTIC VALUE OF BORIC ACID.*

BY EDMUND ANDREWS, M.D., LL.D.

PROFESSOR OF CLINICAL SURGERY IN THE CHICAGO MEDICAL COLLEGE.

The real antiseptic value of boric acid has been a subject of considerable dispute. Owing to the objectionable odor of iodoform, and its irritant effect on the skin, many have tried to substitute boric acid in its place. The published reports about the latter are very conflicting. Some assign to it decided antiseptic virtues, while others claim, on the basis of experiments, that it is not antiseptic at all, and that bacteria will flourish in a saturated solution of it. Curiously enough both these contradictory assertions are in some sense true. Boric acid is so decidedly anti-putrefactive that it is constantly sold and successfully used to preserve fresh meat for market, and a piece of muscular tissue immersed in a saturated solution of it will remain free from odor or softening for weeks, and, probably, for years; yet in that same solution a few living micrococci may sometimes be found, and at least one species of mycelium sprouts freely on the immersed muscular specimen. As boric acid on account of its cleanliness, its freedom from odor, and its absolute safety, is very convenient for extemporaneous use, I have made the following experiments with solutions of it prepared with the hydrant water from Lake Michigan, just as one would find it convenient to do in visiting patients in any of the Lake Shore towns. The water of the streams and wells of adjacent regions does not differ enough from that of Lake Michigan to materially change the results.

It will be noticed that while others allege that they have found the micrococci living in saturated solutions, I was unable to discover any in solutions stronger than one per centum. A saturated solution of boric acid in the water of Lake Michigan at the temperature of 70° Fah., contains about 4 per centum of the acid. The experiments were made in six-ounce bottles of water containing the respective percentages of boric acid mentioned at the head of the columns.

In each bottle was suspended about two drams of fresh pork muscle. Bottle No. 1 contained simple hydrant water; No. 2, one per centum solution of boric acid; No. 3, two per centum solution; No. 4, three per centum solution; No. 5, a saturated solution; No. 6, a solution of what is called *glycerol* of boro-glyceride, that is to say, the solution experimented on contained 4 per centum of boric acid, 12 per centum of glycerine, and 84 per centum of water.

From these experiments it is evident that the saturated solution of boric acid in ordinary use among us is abundantly antiseptic, so far as preventing putrefaction is concerned, but that it does not prevent the growth of mycelium of, at least, some species, and probably does not prevent the development of every species of bacteria and micrococci. We may conclude, therefore, that while boric acid, in all probability, is a good antiseptic for practical purposes, we have not a certainty that every injurious microörganism will be destroyed by it. In short, it lacks the all-conquering energy of the corrosive sublimate solutions. We may conclude with reasonable certainty that, used in a dry form to pack foul ulcers and septic wounds, it will be of decided value on account of not only its antiseptic power, but also its freedom from any caustic or irritating qualities. Decided power is assigned to it when packed into the urethra in dry form to arrest recent attacks of gonorrhœa, as detailed in a recent article by Dr. Joseph Zeissler, of this city.[1]

It will be observed that in the sixth experiment the presence of glycerine seemed to increase the efficiency, so that even the mycelium did not appear. My final conclusion is that for a multitude of purposes boric acid is a very valuable article and convenient for use on account of its cleanliness, freedom from odor, and the total absence of any caustic or irritant qualities, but that in the present state of our knowledge it is not safe to rely upon it exclusively in cases which demand the most decisive and energetic antiseptic qualities. In short, where life is at stake, and may be dependent, to a great extent, on the energy of the antiseptic, it is better at present not to repose too implicit a confidence in boric acid.

6 Sixteenth Street.

Table of Experiments to determine the antiseptic value of Boric Acid by suspending two drachms of fresh muscle in extemporaneous solutions of the Acid in Lake Michigan hydrant water—six ounces to each bottle.

| Days. | Experiment No. 1. Hydrant water alone. | Exp. No. 2. One per cent. of Boric Acid. | Exp. No. 3. Two per cent. of Boric Acid. | Exp. No. 4. Three per cent. of Boric Acid. | Exp. No. 5. Saturated solution Boric Acid. | Exp. No. 6. Boric acid 4 pr. ct. Glycerine 12 " " Water 84 " " |
|---|---|---|---|---|---|---|
| 1 | Solution turbid. | Meat blanched; solution clear; no smell. | Meat blanched; solution clear; no smell. | Meat blanched; solution clear; no smell. | Meat blanched; solution clear; no smell. | Meat blanched; solution clear; no smell. |
| 2 | Decidedly putrid. | Slightly turbid; no smell. | ditto. | ditto. | ditto. | ditto. |
| 3 | | More turbid; mycelium on the meat; no smell. | " | " | " | " |
| 4 | | No smell; mycelium growing; moving micrococci. | No smell; mycelium appearing; no micrococci. | Mycelium appears; no smell; no micrococci. | Mycelium appeared as in preceding experiment. | " |
| 5 | | ditto. | ditto. | ditto. | ditto. | " |
| 6 | | " | " | " | " | " |
| 7 | | " | " | " | " | " |
| 8 | | " | " | " | " | " |
| 9 | | " | " | " | " | " |
| 10 | | " | " | " | " | " |
| 11 | | " | " | " | " | " |
| 12 | | Swimming micrococci; mycelium abundant; no smell; meat firm. | No micrococci: abundant mycelium; no smell; meat firm. | No micrococci; abundant mycelium; no smell; meat firm. | No micrococci; abundant mycelium; no smell; meat firm. | No micrococci; no mycelium; no smell; meat firm; many boro-glyceride crystals at bottom of bottle. |

[1] Zeissler: A Novel Method of Treating Gonorrhœa. Western Medical Reporter, Jan., 1889.

